# Stereotactic ablative body radiation therapy for tumors in the lung in octogenarians: a retrospective single institution study

**DOI:** 10.1186/1471-2407-14-971

**Published:** 2014-12-17

**Authors:** Nathan A Cannon, Puneeth Iyengar, Hak Choy, Robert Timmerman, Jeffrey Meyer

**Affiliations:** Department of Radiation Oncology, University of Texas Southwestern, 5801 Forest Park Rd, Dallas, 75390 Texas USA

**Keywords:** Stereotactic ablative therapy, Geriatric, Lung cancer, Radiation therapy

## Abstract

**Background:**

Treatment of cancer in the lung in octogenarians is limited by their health and functional status. Stereotactic ablative radiotherapy is an established noninvasive treatment option for medically inoperable patients, with a toxicity profile that may be more tolerable in elderly patients.

**Methods:**

Patients more than 80 years old treated with stereotactic ablative radiotherapy for malignant tumors in the lung between January 2007 and August 2012 at a single institution were identified and retrospectively analyzed for toxicity and survival.

**Results:**

Thirty patients were identified with a total of 32 lesions treated. Patients ranged in age from 80.8 to 90.7 years old (median 84.9) at the time of treatment. Twenty patients had ECOG performance status 0–1, and 10 had performance status 2–3. Stage distribution at treatment was: stage I (20 patients), stage III (1), stage IV (1), and 8 recurrent tumors. Patients were treated to a median total dose of 54 Gy in 3 fractions (range 20–60 Gy in 1 to 5 fractions). Median follow up was 13 months (range 2–60 months). Fifteen patients were still living at last review. There was one failure in field and one failure in the same lobe that was treated. One patient died with progressive regional disease, and four died of progressive metastatic disease. Three patients had late grade 3 pulmonary dyspnea with no grade 4 or 5 toxicities. One patient had late grade 2 pneumonitis, and 3 patients had late grade 1 pneumonitis. Three patients had grade 1 chest wall pain.

**Conclusions:**

Octogenarians tolerated ablative treatment with minimal toxicity. Stereotactic ablative body radiotherapy is an option to consider in treatment of elderly patients.

## Background

The ability to treat elderly patients with cancer is complicated due to the increasing comorbidities and decreasing functional status associated with aging [1]. Whether or not age is an independent predictor of poor treatment tolerance remains an area of controversy. For elderly patients treated for lung cancer, declining health means that they may not be candidates for radical surgery and must turn to other options including radiation therapy for management of their localized disease.

For those patients that are not candidates for surgery, conventionally fractionated radiation therapy (e.g., 2 Gy per treatment for 30 treatments) has been associated with poor survival [2, 3]. While the efficacy of radiation treatments does not inherently decline with age [4], targeted tumor control with conventional radiation therapy is significantly inferior to that achieved with lobectomy or sublobar resection [5]. Thus, there is significant interest in alternate treatment options in this group of patients. A variety of thermal ablation techniques are currently used to treat patients with primary and secondary lung cancers occurring in medically inoperable patients [6]. Stereotactic ablative radiotherapy (SABR), also known as stereotactic body radiation therapy (SBRT), is another treatment modality that utilizes advanced planning and image-guidance technologies to deliver potent doses of radiation to discrete tumors in a short treatment course. The dose potencies delivered with SABR are dramatically higher than traditional, conventionally fractionated radiation therapy (e.g., 2 Gy per day given 30 times). A phase II study established SABR as an effective non-surgical therapy for selected patients with medically inoperable early-stage lung cancer, with local control and survival results considerably superior to those historically seen with conventional radiation. In this study, the median age of patients was 72 years [7]. Since SABR is a relatively new treatment modality, there are few studies examining its tolerability and oncologic outcomes in significantly older patients.

We retrospectively examined the experience of patients more than 80 years old treated for tumors in the lung with SABR at a single institution to determine how well these patients tolerated treatment in terms of toxicity following treatment. We also examined the disease free and overall survival of these patients.

## Methods

Between 1/1/2007 and 8/31/2012, 33 patients were treated with SABR to the lung at the University of Texas Southwestern Medical Center who were over the age of 80 (age range 80 to 90 years old) at the time of their first SABR treatment. All patients were either not candidates for lobectomy or declined surgery. Three patients had no follow-up at our institution after treatment and were excluded from the analyses. Comorbidities and demographics are listed in Table 1. Comborbidity definitions are based on previous diagnoses, treatments, or events. Pulmonary comorbidities include emphysema, COPD, and asthma. Cardiac disease included CHF, atrial fibrillation, and a history of myocardial infarction. Hepatic disease includes viral hepatitis and cirrhosis. Patients were evaluated retrospectively as part of an Institutional Review Board approved research review. Patients were staged based on AJCC version 7 based on the stage of disease at the time of presentation for SABR. Patients with recurrent disease were considered to have stage IV disease except for 2 patients with bronchoalveolar carcinoma who had previously resected lesions who were classified as stage I. Tumor location, size, and outcomes are reported in Table 2.

**Table 1:** **Demographics and comorbidities of patients**

		Number of patients
Gender	Male	12
	Female	18
Performance status	0	9
	1	11
	2	8
	3	2
Comorbidities	Pulmonary	15
	Hypertension	16
	Hyperlipidemia	10
	Hypothyroidism	6
	Cardiac	8
	Renal	2
	Cerebrovascular disease	5
	Hepatic	1
	Cancer (not lung)	9
CCI	1	3
	2	8
	3	12
	4	6
	5	1

CCI is Charlson commorbidity index.

**Table 2:** **Summary of patient presentation and outcome**

Patient	Location	Size	Central	Stage	Survival	Status
1	LUL	2.9		IA	Alive	Hillar failure, to hospice
2	LUL	1.5		IA	Alive	No evidence of disease
3	LUL	2.8	Yes	IA	Alive	No evidence of disease
4	RUL	3.5		IA	Alive	No evidence of disease
5	LLL	2.3		IA	Alive	No evidence of disease
6	LUL	2.2		IA	Alive	No evidence of disease
7	LUL	2.3		IA	Deceased	Hillar and contralateral failure, hospice
8	LUL	2.4	Yes	IA	Deceased	No evidence of disease
9	LUL	1		IA	Deceased	No evidence of disease
10	LUL	5.5	Yes	IB	Alive	No evidence of disease
11	RUL	4		IB	Alive	No evidence of disease
12	RUL	4.7	Yes	IB	Alive	No evidence of disease
13	LUL	3		IB	Alive	No evidence of disease
14	LLL	3.6		IB	Alive	No evidence of disease
15	RML	4.5	Yes	IB	Alive	No evidence of disease
16	RLL	4.3		IB	Deceased	Brain metastases, whole brain radiation
17	RML	3.9		IB	Deceased	No evidence of disease
18	RLL	3.4		IB	Deceased	No evidence of disease
19	LUL	3.9	Yes	IB	Deceased	No evidence of disease
20	RLL	3.1		IB	Deceased	No evidence of disease
21	LUL, LUL	1.8, 1.4		IA	Alive	No evidence of disease
22	RUL	3.2		IA	Alive	Failure in the same lobe, SABR
23	RUL	2	Yes	III	Alive	No evidence of disease
24	LUL	2		IA	Deceased	Malignant pleural effusion, hospice
25	LUL	2.2		IA	Deceased	Failure at primary and supraclavicular, SABR and tarceva
26	RLL	6.6		IB	Deceased	No evidence of disease
27	RLL	3		IV	Deceased	No evidence of disease
28	RLL	2.3		III	Deceased	Failure in abdominal soft tissue, tarceva
29	LLL	2.3		IV	Deceased	No evidence of disease
30	RUL, RML	4, 1.5		IV	Deceased	Distant failure in bone, SABR to bone

Therapy at recurrence is noted.

For SABR treatments, patients were immobilized within a large vacuum pillow inside either a commercially available Elekta Stereotactic Body Frame® (Elekta, Crawley, United Kingdom) or a larger carbon fiber body frame. Abdominal compression was applied if tumor motion was greater than 1.0 cm in any direction based on fluoroscopy prior to obtaining CT simulation. Maximum intensity projection (MIP) images from a 4-dimensional CT were registered with the simulation CT scan. The internal target volume (ITV) was defined using MIP images or a Boolean union of inspiration and expiration phases. The planning target volume (PTV) was generated through a 0.5 cm expansion of the ITV in all directions. A treatment plan was generated using 7–13 non-coplanar, non-opposing beams using Pinnacle planning software (Phillips, Amsterdam, Netherlands). Treatment was delivered in 1 to 8 fractions over 1 to 22 days. Prescribed doses ranged from 20 to 60 Gy. (Table 3) Doses were prescribed to the isodose shell covering the PTV (median: 77%, range: 69-97%). One patient was treated to 60 Gy in 3 fractions pre-heterogeneity correction, which is similar to the 54 Gy in 3 fractions with heterogeneity correction. Total dose and fractionation were determined based on the site of the disease (central versus peripheral), stage, trial participation, as well as nearby organ at risk tolerability as determined by the treating physician. Patients were generally treated every other day.

**Table 3:** **Dose fractionation summary**

Total dose	Fractions	Dose per fraction (Gy)	Number of patients
20	3	6.67	1
30	5	6	1
34	1	34	1
35	5	7	1
40	5	8	1
48	8	6	1
50	5	10	5
53	5	10.5	1
54	3	18	13
55	5	11	1
60	3	20	1
60	5	12	7

Follow-up generally consisted of physical examination and CT scan every 3 months for the first year followed by continued exams with CT scans every 6 months. If there were suspicious findings including increase in size of post-radiation scarring or the development of a new nodule or enlarged lymph node, a PET-CT was performed, and FDG-avidity was considered evidence of recurrent disease. If there was still doubt, a biopsy was performed. The Common Terminology Criteria for Adverse Events version 4.0 was used to evaluate toxicity. Dyspnea was defined as shortness of breath with exertion which was persistent. Pneumonitis was defined as shortness of breath with associated symptoms such as fever or cough which resolved after treatment with steroids.

Overall survival was calculated using the Kaplan-Meier methods. Comparisons between groups were made using the log-rank method. Results were considered significant with a P value <0.05. P values were derived from univariate analysis. SPSS version 21 was used for all statistical analyses. (SPSS Inc., Chicago, Illinois, USA) Survival was measured from the first treatment with SABR.

This study was approved by the University of Texas Southwestern Institutional Review Board.

## Results

Between 1/1/2007 and 8/31/2012, 33 patients who were over the age of 80 at the time of first treatment underwent SABR. Three patients had no follow-up after their last treatment and were excluded from further review, leaving 30 patients in the final analyses with median age of 84.9 years at the time of first treatment. Of the 30 patients eligible for the study, 28 are known to have had a biopsy proven nonsmall cell lung carcinoma. One patient had metastatic renal cell carcinoma to the lung treated with SABR. One patient had a biopsy at an outside hospital, and the reports were not available for review but were noted to be nonsmall cell lung carcinoma in the patient’s chart. A total of 32 lesions were treated. Two patients were treated to two isocenters: one with metastatic renal cell and the other with bronchoalveolar carcinoma.

Twenty patients had stage I non-small cell lung cancer (9 stage IA and 11 stage IB). Nine patients were treated at the time of recurrence of non-small cell lung cancer. Two of these patients had bronchoalveolar carcinoma and were treated to new sites of disease (patients 21 and 24, see Table 2). Patient 22 had stage IB carcinoma of the lung but was not a surgical candidate. This patient was initially treated with chemoradiotherapy but had progression at the primary site, which was subsequently treated with SABR. Patient 23 had stage III (T2N2M0) non-small cell lung cancer treated with surgical resection. This patient was treated with carboplatin and paclitaxal with bevacizumab at the time of first progression in the mediastinum. The patient recurred a second time following chemotherapy, this time in the contralateral lung. The site of second recurrence was treated with SABR. Two patients (patient 25 and 26) had a previous resection and were treated with SABR at the time of recurrence in an isolated nodule. Patient 27 had stage IV disease previously treated with carboplatin and paclitaxal followed by navelbine and later topotecan. This patient was treated to a single progressive lesion in the lung. Also, 2 patients were treated to isolated nodules in conjunction with concurrent chemoradiation to the primary and mediastinum (patients 28 and 29). Patient 30 had metastatic renal cell carcinoma and was treated to 2 sites of metastatic disease.

Nine patients had a previous diagnosis of non-lung cancer. Of these patients, 7 had been previously treated and had control of the cancer. One had recently been diagnosed with prostate cancer and declined treatment; the lung cancer was discovered on staging workup. The final patient had metastatic renal cell carcinoma to the lung and abdominal lymph nodes, all of which were treated with SABR.

**Figure 1:**
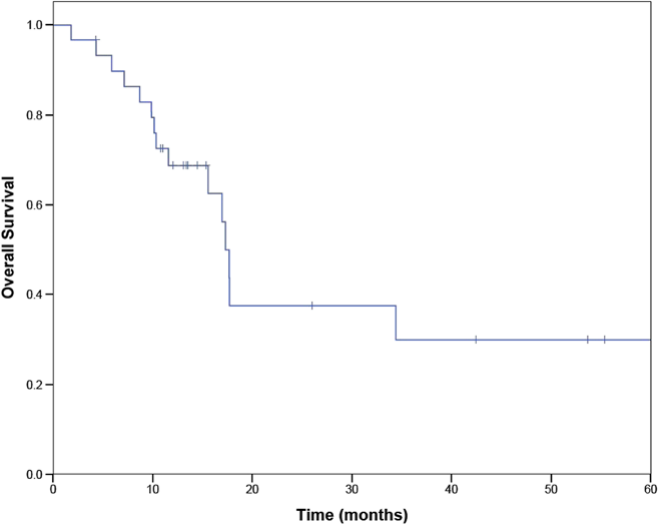
**Overall survival of octogenarians treated with SABR.**

**Figure 2:**
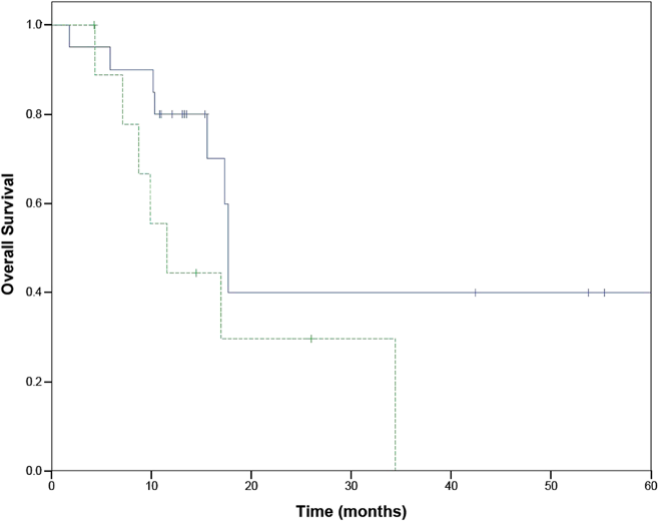
**Overall survival of patients with early and late stage lung cancer.** Stage 1 in solid line. Recurrent or advanced stage disease in dashed line.

**Figure 3:**
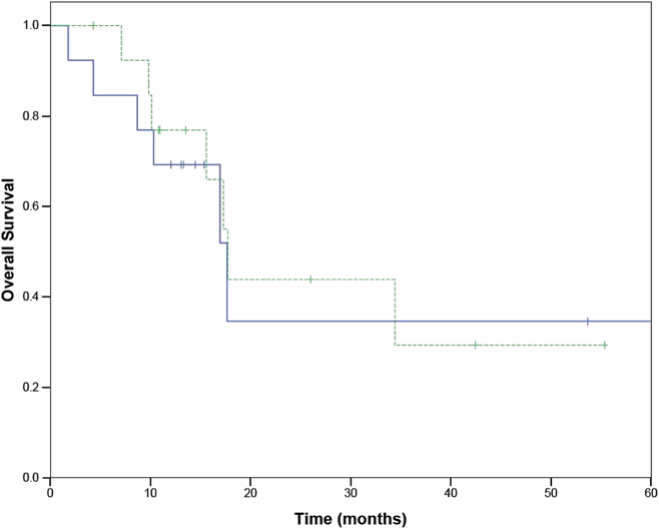
**Survival of early stage patients in 3 and 5 fractions of SABR.** 3 fractions in solid line. 5 fractions in dashed ling.

Nineteen patients had ECOG performance status 0–1 at time of treatment, and 11 had performance status 2–3. The distribution of the stage of the patients is shown in Table 2. Seven of the tumors were in a central location (within 2 cm of the proximal bronchial tree), and each of these were treated with at least a 5 fraction regimen (total dose 40–60 Gy).Median follow up was 13 months with a range from 2 to 61 months. There were no hospitalizations within 30 days of completing treatment. Actuarial survival at one year was 69%. (Figure 1) Fifteen patients remained alive at the time of last follow-up. When patients were split into stage I versus recurrent or advanced stage, the median survival of patients with stage I disease was 17.7 months (95% confidence interval 16.1-18.5 months), and the 2 year Kaplan-Meier survival was 36%. The median survival of patients with stage III-IV disease was 11.6 months (95% confidence interval 7 to 17 months). (Figure 2) The difference in overall survival between early and recurrent or advanced stage patients was not statistically significant. In patients with early stage lung cancer treated with 3 or 5 fractions of radiation, there was no difference in overall survival based on the fractionation scheme (Figure 3)

.

Three patients failed in the ipsilateral lung. Patient 24 treated for bronchoalveolar carcinoma developed a malignant pleural effusion and went to hospice 14 months after treatment. The second patient (patient 22) was treated for recurrent disease and developed a second tumor outside of the radiation field in the same lobe that was treated. This recurrence was treated with SABR one year after the initial treatment which was complicated by pneumonitis. The third patient (patient 25) had previously been treated with surgical resection followed by SABR at the time of recurrence. He developed progression of the primary lesion. This patient died 26 months after the initial treatment. Another patient had progression in the lungs, but this was a site previously treated with conventional radiation contralateral to the SABR treatment. Four of the patients who died had known metastatic disease at the time of death and died without evidence of local progression. One patient died of malignant melanoma known at the time of initial treatment. Two patients died of other causes (pneumonia and cardiac arrest). There were no treatment related deaths. The cause of death was unknown in 7 patients.

Patients developed toxicity related to respiratory difficulties and chest wall pain. The most common late side effect was dyspnea. Three patients developed grade 1 dyspnea. Three patients developed grade 3 dyspnea 1–12 months after completing treatment. One patient developed grade 2 pneumonitis four months after finishing treatment which resolved with a short course of steroids. Three patients developed grade 1 chest wall pain 1 to 9 months after treatment. Two of these patients received 60 Gy in 5 fractions and one received 54 Gy in 3 fractions. In total, 7 patients experienced late toxicity associated with SABR. In patients receiving 54 Gy in 3 fractions (14 patients including the patient treated with 60 Gy in 3 fraction pre-heterogeneity correction), 2 patients developed grade 3 dyspnea compared to 1 patient with grade 2 and 1 patient with grade 3 dyspnea who were treated to 50 to 60 Gy in 5 fractions (13 patients).

## Discussion

The tolerance of the most elderly patients to intensive cancer therapies, irrespective of comorbid conditions, is an area of controversy. Laboratory studies have not definitively shown a relationship between age and increased sensitivity to radiation for skin, vascular smooth muscle cells, and chromosomal breaks [8–10]. Clinical reports have shown conflicting data, but the available evidence suggests that older patients with lung cancer undergoing concurrent chemotherapy and radiation therapy have higher rates of pneumonitis and hematologic toxicity compared to younger patients [11].

SABR is now an established option for the management of early-stage lung cancer in patients who are not candidates for conventional surgery because of medical comorbidities. Although many of these patients are elderly, the safety and efficacy of high-dose focal irradiation in octogenarian and older patients is not well defined. One of the hallmarks of SABR is delivery of radiation doses (typically in the range of 8–20 Gy per fraction) that are significantly higher than those delivered with more conventional fractionated irradiation. Advances in treatment planning and delivery have allowed the highest doses to be conformally confined to the tumor volume, but there is nonetheless a zone of intermediate falloff dose within normal tissue that may be poorly tolerated in elderly tissues.

Many studies have looked at the feasibility of surgical treatments in octogenarians who are fit for surgery. Traditional open surgeries can be tolerated in carefully selected octogenarians [12, 13]. More recently, video assisted thoracoscopic surgery (VATS) has been found to be tolerable with similar rates of perioperative mortality and survival to open surgery in octogenarians [14–18]. VATS has also been shown to have fewer complications following open surgeries [19]. Surgery has remained the primary modality of treatment in the elderly and octogenarians for patients with early stage lung cancer due to its superior overall survival compared to conventional radiation or chemotherapy [5]. Unfortunately, many elderly patients with lung cancer have multiple comorbidities which make them poor candidates for surgery, with 88% having at least 1 and 54% having 3 or more comorbidities [1].

Despite the lack of evidence for its benefits in this population, SABR is becoming more common in the elderly and in octogenarians [20]. Preliminary data indicate that age is not a significant predictor of overall survival in elderly patients being treated with SABR, but worse performance status is a predictor of poor outcome [21, 22]. Recent studies of SABR in octogenarians with early stage lung cancer showed that patients tolerated a 5 fraction regimen well with minimal side effects (Table 4) [21, 23–26]. Takeda et al. showed a 3 year overall survival and lung cancer specific survival of 53.7 and 70.8% respectively. The majority of patients in our study were treated with a 3 fraction regimen which is common in the United States. The patients with early stage disease in our study had 2 year actuarial survival of 41%. In our study, there was no difference in survival between patients receiving treatment in 3 or 5 fractions.

**Table 4:** **Previous studies of SABR in the elderly**

Study	Median age (Minimum)	Patients	Grade 3 toxicity (%)	Dose (Gy)	Fractions
Hasbeek	79(75)	193	7	60	3-8
Sugane	82(80)	28	0	52.8-72	4-9
Samuel	82(75)	46	0	48-60	3-5
Takeda	83(80)	109	6	40-50	5
Van der Voort	82(80)	38	5	48-60	3-6
Present	85(80)	30	10	20-60	1-8

With toxicity being a major concern in elderly patients, limiting the side effects of treatment is critical. Samuel et al. examined toxicity in elderly patients treated with SABR with 3 or 4 fractions [24]. They did not observe any grade 3 toxicity. All of the studies examining SABR in elderly patients have shown relatively low rates of grade 3 toxicity ranging from 0-10%, but they did not compare toxicity rates in patients based on fractionation schedule. Overall, these rates of toxicity are similar to those seen for younger patients treated with SABR [7]. In our study, there was no difference in the rates of dyspnea or pneumonitis when comparing treatments of 3 or 5 fractions. This is the first study to include both treatments of 3 and 5 fractions and compare them directly for toxicity. However, there is not sufficient power in our study to detect small differences in side effects. These issues could be examined in future studies.

Mounting evidence indicates that age alone should not be a significant deciding factor in whether or not therapies with curative intent are offered to patients with lung cancer. In patients with locally advanced lung cancer, Domingues et al. showed that definitive therapy with radiation or concurrent chemotherapy and radiation in elderly patients with a median age of 71 (range 65–90) significantly increased the overall survival of patients compared to palliative care alone, and this survival advantage remained for those patients over the age of 75 [27]. Blanchard reviewed the results of patients over the age of 80 and concluded that patients would benefit from definitive therapy with radiation or chemotherapy [28]. Our study showed that patients with recurrent or advanced cancer tolerated treatment well with minimal side effects and obtained good local control based on 7 patients with stage III or IV disease. While 12 month survival was 44%, and the median survival was 11.6 months. Control of disease at the site of the lung mass could be beneficial in these patients in terms of decreased symptoms of lung collapse or obstructive pneumonia. This is the only study which included patients with advanced stage disease who were treated with SABR. While this included only 10 patients, the treatment was well tolerated, and there was one in-field failure. None of the patients died of treatment related toxicity or local progression, indicating that the treatments were well tolerated and effective in preventing complications of progressive disease at the site of treatment. Further investigation of the benefits of these treatments is warranted.

## Conclusion

This study shows that SABR, including 3-fraction and 5-fraction treatments, is a viable treatment option in octogenarians with cancer in the lung who are not candidates for surgical resection. Further research should be conducted to ascertain the full benefits of treatment in these patients in terms of palliative benefit and improvements in survival. The local control of the targeted disease is very good with one in field failure in this study. Treating patients with 3 or 5 fractions are both viable options resulting in acceptable toxicity while affording impressive rates of overall survival.

## Abbreviations

ECOG: Eastern Cooperative Oncology Group
Gy: Grey
ITV: Internal target volume
MIP: Maximum intensity projection
PTV: Planning target volume
SABR: Stereotactic ablative radiotherapy
SBRT: Stereotactic body radiation therapy
VATS: Video assisted thoracic surgery.
